# Supplementary figures and images for: Utility of data from the Danish National School Test Program for health research purposes: Content and associations with sociodemographic factors and higher education
Source: PLoS One. 2024 May 1;19(5):e0302472. doi: 10.1371/journal.pone.0302472 (PMC11062538; doi:10.1371/journal.pone.0302472)

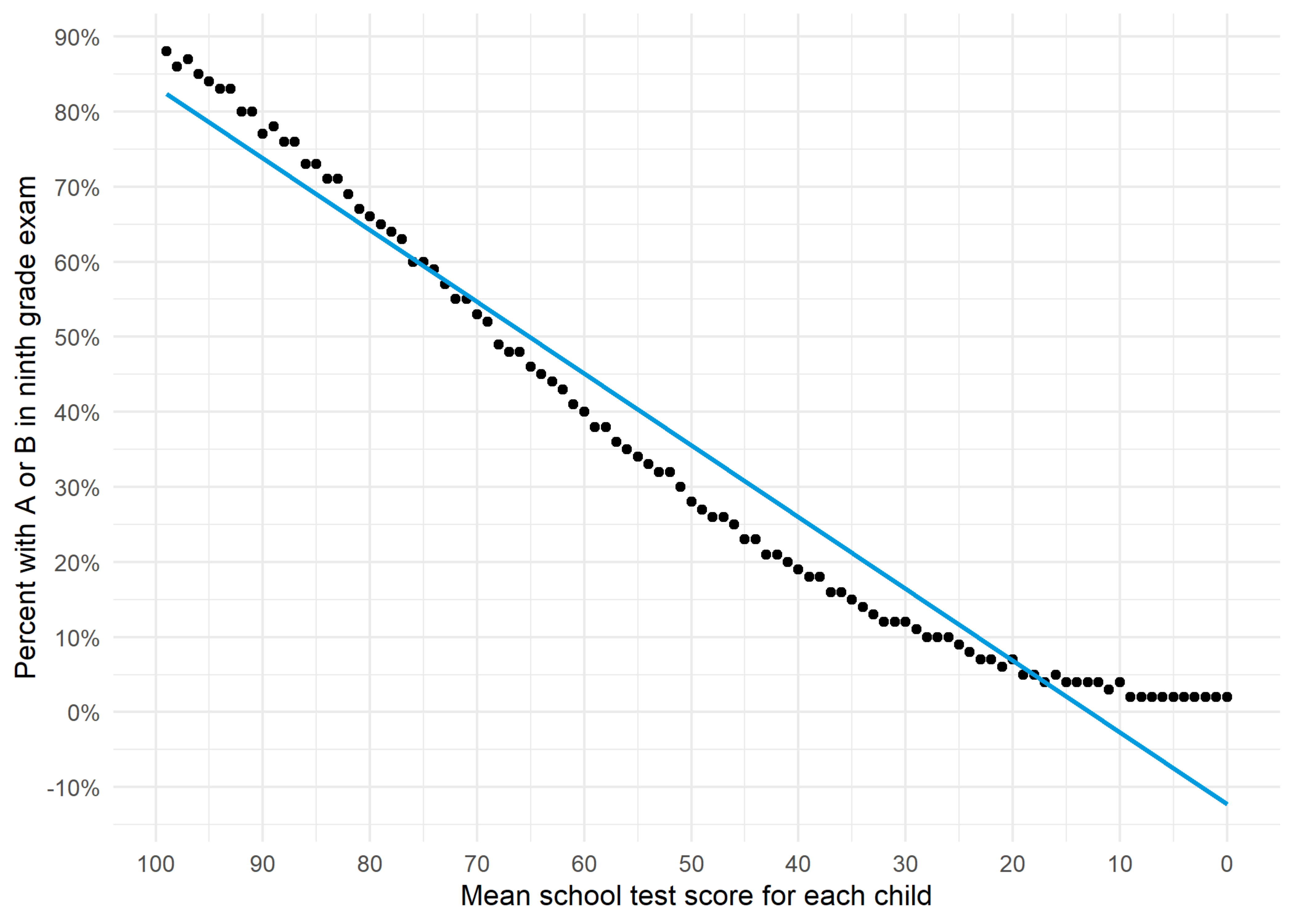

Supplement: S1 Fig — Mean test scores were rounded to the nearest integer. A decrease of 1 point in the test scores was associated with a decrease of 0.95% (95% CI: 0.93%; 0.97%) in obtaining B or higher in the ninth-grade final exam. The intercept of the linear regression was 82.4% (95% CI: 81.2%; 83.5%). Although the data points did not have a linear relationship and the linear regression model predicted negative percentages for test scores below 13 points, the model predicted fairly well for test score points between 20 and 70. (PDF) [file pone.0302472.s001.pdf]

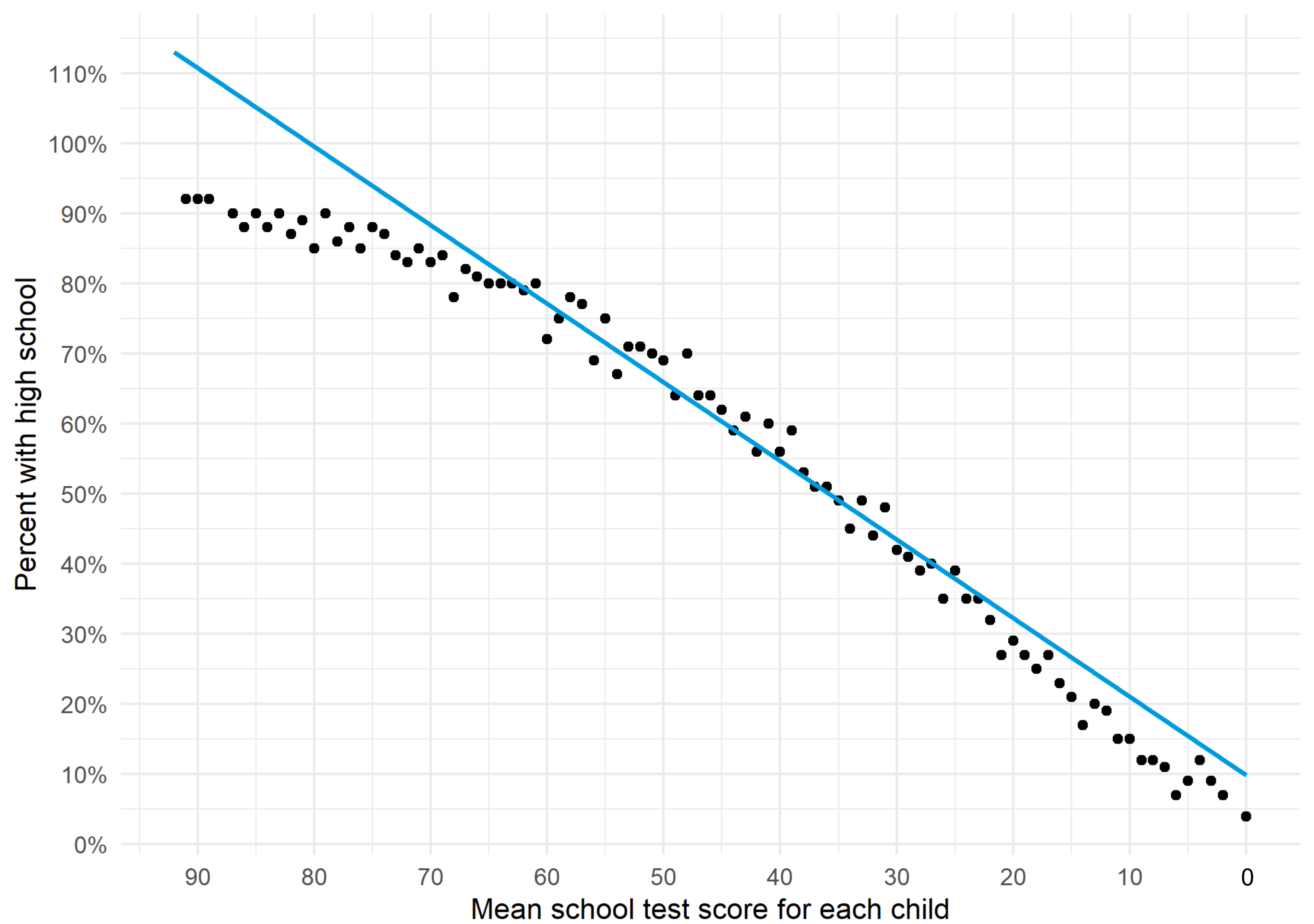

Supplement: S2 Fig — Highest educational level was assessed for 50,650 children with an 8th grade reading test in 2012 and 49,520 children with a 6th grade mathematics test in 2010. Mean test scores were rounded to the nearest integer. A decrease of 1 point in the test scores was associated with a decrease of 1.03% (95% CI: 1.00%; 1.05%) in completing high school. The intercept of the linear regression was 113.3% (95% CI: 112.0%; 114,7%). Although the data points did not have a linear relationship and the linear regression model predicted percentages above 100% for test scores above 81 points, the model predicted fairly well for test score points between 20 and 70. The following test scores were removed because there were too few children in some groups of education: 2, 89, 93, 94, 95, 96, 97, 98, 99, and 100. (PDF) [file pone.0302472.s002.pdf]
