# Supplementary material for: Utility of data from the Danish National School Test Program for health research purposes: Content and associations with sociodemographic factors and higher education
Source: PLoS One. 2024 May 1;19(5):e0302472. doi: 10.1371/journal.pone.0302472 (PMC11062538; doi:10.1371/journal.pone.0302472)
Supplement: S1 Table — a Scores are divided according to norm-referenced groups. b Information on ninth-grade final exams was obtained for children with school tests in the years 2010–2018. Grades from the Danish scale were converted into European Credit Transfer System grades (ECTS). c Highest educational level within five years after graduating primary and lower secondary school was assessed for 50,650 children with an 8th grade reading test in 2012 and 49,520 children with a 6th grade mathematics test in 2010. Other includes children that completed an education after primary and lower secondary school but did not complete high school. High school or vocational includes children that completed high school and possibly other educations. (DOCX) [file pone.0302472.s003.docx]

|  | **Reading tests (Danish)** | | | | |  | **Mathematics tests** | | | | |
| --- | --- | --- | --- | --- | --- | --- | --- | --- | --- | --- | --- |
| Test Score^a^ | Below average (1-35) | |  | Above average (36-100) | |  | Below average  (1-35) | |  | Above average  (36-100) | |
|  | n | (%) |  | n | (%) |  | n | (%) |  | n | (%) |
| Performance in ninth-grade final exam^b^ |  |  |  |  |  |  |  |  |  |  |  |
| Number of children | 189,910 | - |  | 637,380 | - |  | 147,200 | - |  | 508,360 | - |
| A | 60 | (0.0) |  | 36,180 | (5.7) |  | 240 | (0.2) |  | 45,980 | (9.0) |
| B | 5,130 | (2.7) |  | 132.230 | (20.7) |  | 2,980 | (2.0) |  | 89,740 | (17.7) |
| C | 27,920 | (14.7) |  | 129,280 | (20.3) |  | 18,230 | (12.4) |  | 96,680 | (19.0) |
| D | 43,640 | (23.0) |  | 60,370 | (9.5) |  | 32,560 | (22.1) |  | 39,850 | (7.8) |
| E | 23,980 | (12.6) |  | 9,840 | (1.5) |  | 21,320 | (14.5) |  | 8,040 | (1.6) |
| F/Fx | 2,350 | (1.2) |  | 380 | (0.1) |  | 5,630 | (3.8) |  | 880 | (0.2) |
| Missing | 86,820 | (45.7) |  | 269,110 | (42.2) |  | 66,250 | (45.0) |  | 227,180 | (44.7) |
|  |  |  |  |  |  |  |  |  |  |  |  |
| Highest educational level^c^ |  |  |  |  |  |  |  |  |  |  |  |
| Number of children | 11,390 | - |  | 39.260 | - |  | 35,650 | - |  | 13,870 | - |
| Primary and lower  secondary | 5,450 | (47.9) |  | 6,580 | (16.8) |  | 6,180 | (44.6) |  | 6,160 | (17.3) |
| Other | 2,030 | (17.8) |  | 2,280 | (5.8) |  | 1,940 | (14.0) |  | 2,320 | (6.5) |
| High school or  vocational | 3,910 | (34.3) |  | 30,410 | (77.5) |  | 5,750 | (41.5) |  | 27,170 | (76.2) |
