# Supplementary material for: Utility of data from the Danish National School Test Program for health research purposes: Content and associations with sociodemographic factors and higher education
Source: PLoS One. 2024 May 1;19(5):e0302472. doi: 10.1371/journal.pone.0302472 (PMC11062538; doi:10.1371/journal.pone.0302472)
Supplement: Attachment — Submitted filename: RE.docx [file pone.0302472.s004.docx]

RE: The Danish National School Test Program: Correlates with Sociodemographic Factors

and Prediction of Academic Achievement

The literature review is very light-on. There are many studies on achievement over school career (by level) and the correlations between achievement in mathematics and reading (Reynolds & Walberg, 1992, p. 318; Armor, 2003, p. 33; McNiece, Bidgood, & Soan, 2004, p. 134; Parsons, 2014, p. 36; Sullivan, Parsons, Wiggins, Heath, & Green, 2014, p. 752; Kriegbaum, Jansen, & Spinath, 2015; Ludeke et al., 2021, p. 1083; Marks, 2021). There is a huge academic literature on SES and achievement.

I am not requesting an extensive literature review, but some discussion of the results found in previous studies, which are similar to the those in presented in the reviewed paper: high and possibly increasing within domain temporal correlations and slightly weaker between-domain correlations. In their Discussion section, the authors should attempt to explain why the results are similar, or in the authors’ judgement, different from, comparable studies conducted in other countries.

There are no theoretical explanations in the paper for the patterns observed. Could the authors please discuss their results in relation to the literature on general and specific cognitive abilities or an alternative explanation they find plausible.

According to the title of the paper, it is about sociodemographic factors. My question is, do socioeconomic factors strongly impact on test scores when considering prior test scores. With prior achievement accounting for between 25 and 60% of the variance, I expect the effects of sociodemographic factors to be quite weak.

The paper needs some additional analyses with and without prior test scores vis-a-vis continuous some measures of the socioeconomic characteristics (mother’s and father’s education, and logged family income). The paper is purportedly about socioeconomic factors, but they are hardly discussed.

What are the correlations of test scores with the SES measures? Could continuous measures of father’s and mother’s education, and income be added to eFigure 1 (which should replace Figure3 in the main body). It is not clear if SES correlations are weaker in Denmark than in other countries and although the authors suggest that they are probably are weaker because of a more extensive welfare state. They suggest comparing their findings with similar studies, but they do not seem to do so.

1. I expected to find the regressions in the Supplementary material which back up the claims in the main body of the paper. They don’t seem to be there.
2. Why a B or above cutoff rather than simply Grade 9 test score which is statistically simpler and more interpretable. Why discard all the information in a normally distributed variable and arbitrarily choose a cut-off of B or higher?
3. I find the correlations in eFigure 1 more meaningful to the estimates in Figure 3.
4. The estimates presented in eTable 1 are not how estimates are usually presented. For most readers, much more interpretable estimates would be changes in test scores in metric or standard deviation units for a one-standard deviation increase prior test score. This was done for GPA in the previous paper.
5. There’s no need for confidence intervals in eTable 1. The estimates and two confidence limits are exactly the same. They just clutter the table.
6. The three cited studies (25-27) are not very relevant to the paper. I found the choice of papers a bit odd; all 3 are highly econometric.
7. Relatedly, why is the previous study of Danish National Test Program mentioned in the Discussion? The Introduction should provide a summary of the results and limitations of the previous study. The reference should provide a link to the study (https://childresearch.au.dk/fileadmin/childresearch/dokumenter/2018_1_2.pdf ).
8. The paper would be more interesting if university entrance or tertiary course status were included in the measure of educational attainment, not just completing high school.

Armor, D. J. (2003). *Maximizing intelligence*. New Brunswick and London: Transaction Publishers.

Kriegbaum, K., Jansen, M., & Spinath, B. (2015). Motivation: A predictor of PISA's mathematical competence beyond intelligence and prior test achievement. *Learning and Individual Differences, 43*, 140-148. doi:<https://doi.org/10.1016/j.lindif.2015.08.026>.

Ludeke, S. G., Gensowski, M., Junge, S. Y., Kirkpatrick, R. M., John, O. P., & Andersen, S. C. (2021). Does parental education influence child educational outcomes? A developmental analysis in a full-population sample and adoptee design. *Journal of Personality and Social Psychology, 120*(4), 1074-1090. doi:<https://doi.org/10.1037/pspp0000314>.

Marks, G. N. (2021). Should value-added school effects models include student- and school-level covariates? Evidence from Australian population assessment data. *British Educational Research Journal, 47*(1), 181–204. doi:<https://doi.org/10.1002/berj.3684>.

McNiece, R., Bidgood, P., & Soan, P. (2004). An investigation into using national longitudinal studies to examine trends in educational attainment and development. *Educational Research, 46*(2), 119-136. doi:<https://doi.org/10.1080/0013188042000222412>.

Parsons, S. (2014). *Childhood cognition in the 1970 British cohort study*. Retrieved from London: <https://cls.ucl.ac.uk/wp-content/uploads/2017/07/BCS70-Childhood-cognition-in-the-1970-British-Cohort-Study-Nov-2014-final.pdf>

Reynolds, A. J., & Walberg, H. J. (1992). A process model of mathematics achievement and attitude. *Journal for Research in Mathematics Education, 23*(4), 306-328. Retrieved from <http://www.jstor.org/stable/749308>.

Sullivan, A., Parsons, S., Wiggins, R., Heath, A., & Green, F. (2014). Social origins, school type and higher education destinations. *Oxford Review of Education, 40*(6), 739-763. doi:<https://doi.org/10.1080/03054985.2014.979015>.
