# Supplementary material for: Utility of data from the Danish National School Test Program for health research purposes: Content and associations with sociodemographic factors and higher education
Source: PLoS One. 2024 May 1;19(5):e0302472. doi: 10.1371/journal.pone.0302472 (PMC11062538; doi:10.1371/journal.pone.0302472)
Supplement: Attachment — Submitted filename: Response to Reviewers.docx [file pone.0302472.s005.docx]

Dear Dr. Daniel Ramskov and editorial staff,

Thank you for sending us the comments and feedback from the editors and reviewers. We thank everyone for taking the time to read our manuscript and providing valuable and constructive input. We appreciate the opportunity to revise and resubmit our manuscript, which we believe is improved based on the suggested revisions. Please find below a detailed point-by-point response to all comments (the editors' and reviewers' comments in black, our replies in blue). The page numbers mentioned are based on the clean version of our manuscript.

# Journal Requirements

***Journal Requirement #1:*** When submitting your revision, we need you to address these additional requirements. Please ensure that your manuscript meets PLOS ONE's style requirements, including those for file naming. The PLOS ONE style templates can be found at https://journals.plos.org/plosone/s/file?id=wjVg/PLOSOne_formatting_sample_main_body.pdf
https://journals.plos.org/plosone/s/file?id=ba62/PLOSOne_formatting_sample_title_authors_affaffiliati.pdf

***Response:*** We have updated the manuscript such that it adheres to the formatting guidelines.

***Journal Requirement #2:*** We note that the grant information you provided in the ‘Funding Information’ and ‘Financial Disclosure’ sections do not match. When you resubmit, please ensure that you provide the correct grant numbers for the awards you received for your study in the ‘Funding Information’ section.

***Response:*** We have updated the section ‘Funding Sources’ so that it matches the information provided in the ‘Funding Information’

***Journal Requirement #3:*** Thank you for stating the following in the Competing Interests section: "I have read the journal's policy and the authors of this manuscript have the following competing interests: J.C. has received honoraria from serving on the scientific advisory board of UCB Nordic and Eisai AB, received honoraria for giving lectures from UBC Nordic and Eisai AB, and received funding for a trip from UCB Nordic.".
Please confirm that this does not alter your adherence to all PLOS ONE policies on sharing data and materials, by including the following statement: "This does not alter our adherence to  PLOS ONE policies on sharing data and materials.” (as detailed online in our guide for authors http://journals.plos.org/plosone/s/competing-interests).If there are restrictions on sharing of data and/or materials, please state these. Please note that we cannot proceed with consideration of your article until this information has been declared. 
Please include your updated Competing Interests statement in your cover letter; we will change the online submission form on your behalf.

***Response:*** We have included the updated Competing Interests statement in the revised cover letter.

***Journal Requirement #4:*** We note that you have indicated that data from this study are available upon request. PLOS only allows data to be available upon request if there are legal or ethical restrictions on sharing data publicly. For more information on unacceptable data access restrictions, please see http://journals.plos.org/plosone/s/data-availability" \l "loc-unacceptable-data-access-restrictions.

In your revised cover letter, please address the following prompts:

a) If there are ethical or legal restrictions on sharing a de-identified data set, please explain them in detail (e.g., data contain potentially sensitive information, data are owned by a third-party organization, etc.) and who has imposed them (e.g., an ethics committee). Please also provide contact information for a data access committee, ethics committee, or other institutional body to which data requests may be sent.

b) If there are no restrictions, please upload the minimal anonymized data set necessary to replicate your study findings as either Supporting Information files or to a stable, public repository and provide us with the relevant URLs, DOIs, or accession numbers. For a list of acceptable repositories, please see http://journals.plos.org/plosone/s/data-availability.

We will update your Data Availability statement on your behalf to reflect the information you provide.

***Response:*** We have included an updated data availability statement in the revised cover letter, where we address prompt a).

The statement is as follows:

**Data Availability:** Data were based on Danish national registers, and individual-level data cannot be shared due to national regulations. The data is governed by the Danish Government, Statistics Denmark and the Ministry of Children and Education. Original data are available upon request to the relevant authorities. Additional results are provided in the supplementary material.

Reviewer #1

***Comment 1:*** This study does majorly suffer from two important things: A misguided description of the research literature on academic achievement, including the Danish context. There are many large-scale studies in for example Denmark, Norway and Sweden. Also, the international literature is largely missing.

***Response:***
We apologize for not providing a sufficient description of the research literature and thank the reviewer for listing additional studies.

Our intention with the present paper was to provide a description of this specific data set of test scores from the Danish National Test Program with the purpose of showing the value of the data for health researchers (like ourselves).

Surely, our intention was not to say that educational data (in general) have rarely been used in research before – a statement that we know certainly is not true, given the widespread use of educational data in many other scientific disciplines, including social science, political science etc. Our intention was instead to say that *these specific data* have not been used widely for health research purposes.

However, we realize that this point was not clear from the introduction. We have therefore now revised the introduction to include additional literature, particularly in relation to academic achievement, but also clarified our aims, to set a narrower context for this paper.

In addition, we revised the title of the manuscript to:

“*Utility of data from the Danish National School Test Program for health research purposes: Content and associations with sociodemographic factors and higher education*”.

***Comment 2:*** This study does majorly suffer from two important things: Omitted variable bias. Considerable research shows that socioeconomic and sociodemographic factors are not strongly associated with academic achievement when cognitive ability and conscientiousness (or similar non-cognitive constructs such as academic self-concept and/or self-efficacy) are included in the same multivariate models. Hence, readers cannot have a strong confidence in your regression estimates.

If there are no such variables in your data set you must critically discuss the limitations of your results. This has not been done in the manuscript.

I suggest that you work with the data and include more variables and then re-analyze the revised models. Please do also include some of the studies that I list below.

***Response:*** We agree that omitted variable bias is a weakness of the study, and we thank the reviewer for pointing this out. Unfortunately, our data does not contain variables on cognitive abilities and non-cognitive constructs, meaning that we cannot make multivariable models that consider their influence.

Additionally, we want to emphasize that the aim of our study is to describe the data available in the Danish National School Test Program and show how the test scores differ according to sociodemographic characteristics (which is why all estimates are presented as simple bivariate associations). Our intention was not to conduct a causal analysis of the factors that influence academic achievement. We realize that this was not clear. We have therefore revised the introduction of the paper (as per our response to comment #1) and as suggested, we have now addressed the limitations of our results in relation to omitted variable bias in the discussion section. We thank the reviewer for highlighting this important issue.

Please see discussion, page 16:

*There are numerous theoretical explanations for socioeconomic inequalities in academic achievement, including cognitive abilities and personality traits such as conscientiousness.[2, 28, 29] The substantial association between socioeconomic factors and academic achievement found in this study, does not address underlying factors such as prior test scores and cognitive abilities, that likely reflect an interaction between gene and environmental factors which would predict both school performance and socioeconomic status.[29, 30]* *It was not the focus of the this mainly descriptive study to explain the observed socio-economic differences.*

Reviewer #2

***Comment 1:*** The literature review is very light-on. There are many studies on achievement over school career (by level) and the correlations between achievement in mathematics and reading. There is a huge academic literature on SES and achievement.

I am not requesting an extensive literature review, but some discussion of the results found in previous studies, which are similar to the those in presented in the reviewed paper: high and possibly increasing within domain temporal correlations and slightly weaker between-domain correlations. In their Discussion section, the authors should attempt to explain why the results are similar, or in the authors’ judgement, different from, comparable studies conducted in other countries.

***Response:*** We apologize for not including sufficient literature in our study. This issue was also brought up by reviewer 1, and we refer to our response to their comment #1 in relation to revisions done in the introduction.

Furthermore, our results are now discussed more in the context of the existing literature, see discussion page 17:

*We found moderate to high correlations between prior and later test results, suggesting that prior test scores can serve as reliable indicators of performance in later tests in The Danish National School Test Program. The correlation was strongest for tests that were temporally closer and for tests within the same subject. Nonetheless, there were still moderate correlations between scores in mathematics and reading, which may be explained by a general interest in learning. Similar results were found in previous studies.[5, 29, 31, 32]*

***Comment 2:*** There are no theoretical explanations in the paper for the patterns observed. Could the authors please discuss their results in relation to the literature on general and specific cognitive abilities or an alternative explanation they find plausible.

***Response:*** We thank the reviewer for this important suggestion. We have now included a section in the discussion about theoretical explanations for the relationship between socioeconomic factors and academic performance.

Discussion, page 16:

*The magnitude of the difference in test performance between levels of parental socioeconomic characteristics was high in our study (up to 27 out of 100 points). Like previous studies, we found that low socioeconomic status of parents was associated with poorer school performance in the children.[2, 26, 27] There are numerous theoretical explanations for socioeconomic inequalities in academic achievement, including cognitive abilities and personality traits such as conscientiousness.[2, 28, 29]*

***Comment 3:*** According to the title of the paper, it is about sociodemographic factors. My question is, do socioeconomic factors strongly impact on test scores when considering prior test scores. With prior achievement accounting for between 25 and 60% of the variance, I expect the effects of sociodemographic factors to be quite weak.
The paper needs some additional analyses with and without prior test scores vis-a-vis continuous some measures of the socioeconomic characteristics (mother’s and father’s education, and logged family income). The paper is purportedly about socioeconomic factors, but they are hardly discussed.

***Response:*** We agree with the reviewer that the effects of sociodemographic factors would likely be small if we included prior achievement.

However, since the scope and aim of our study is to describe the data in the Danish National School Test Program, and not to conduct a causal analysis of academic achievement, we think that it is more appropriate to leave out the multivariable regression models with prior test scores and socioeconomic factors. We apologize for any confusion regarding the aim/purpose of our study, and we hope that it is now more clearly conveyed.

We have included the following in the discussion, page 16:

*The substantial association between socioeconomic factors and academic achievement found in this study, does not address underlying factors such as prior test scores and cognitive abilities, that likely reflect an interaction between gene and environmental factors which would predict both school performance and socioeconomic status.[29, 30] It was not the focus of the this mainly descriptive study to explain the observed socio-economic differences.*

***Comment 4:*** What are the correlations of test scores with the SES measures? Could continuous measures of father’s and mother’s education, and income be added to eFigure 1 (which should replace Figure3 in the main body). It is not clear if SES correlations are weaker in Denmark than in other countries and although the authors suggest that they are probably are weaker because of a more extensive welfare state. They suggest comparing their findings with similar studies, but they do not seem to do so.

***Response:*** First, we thank the reviewer for the suggestion to add parental education and income to the new Fig 3 (what was S1 Fig before). We have followed the suggestion but find that this revised version (see inserted below) is less readable than the original version of the figure and would therefore prefer to keep the original version. If the Editor disagree with us on this assessment, we will change the figure as suggested. Additionally, we point out that the association between parental educational level and income and child test scores is provided in Table 3.

Second, we agree with the reviewer that it is not clear from our findings whether SES correlations are weaker in Denmark than in other countries. We believe that it is not straightforward to compare the magnitude of the effect of SES on academic performance with studies from other countries, since our findings on SES are not adjusted with the relevant covariates (cognitive abilities, non-cognitive constructs, and prior test scores, etc.). We have now changed the section such that we no longer suggest comparing our findings to similar studies from other countries.

***Comment 5:*** I expected to find the regressions in the Supplementary material which back up the claims in the main body of the paper. They don’t seem to be there.

***Response:*** All estimates presented in this paper are from bivariate models – i.e. regressions do not contain any additional information than what we are providing. We are not sure which regression results the reviewer is requesting. We kindly ask the reviewer to elaborate on this point.

***Comment 6:*** Why a B or above cutoff rather than simply Grade 9 test score which is statistically simpler and more interpretable. Why discard all the information in a normally distributed variable and arbitrarily choose a cut-off of B or higher?

***Response:*** The grades from the ninth-grade exam were converted from the Danish scale to the European Credit Transfer Systems grades (ECTS) which is a discrete scale with six levels (A, B, C, D, E, F). We believe that treating discrete ECTS grades as a normally distributed variable is not appropriate. We use the cut-off of B or higher in the regression analysis because we want an interpretation that provides good discrimination and does not depend on all six levels of grades.

This is now clarified in the manuscript on page 7:

*Information on ninth-grade exam results in reading (Danish) and mathematics was obtained up until 2019[19] and converted into European Credit Transfer System grades (ECTS), which consists of six levels (A, B, C, D, E, F).[24]*

***Comment 7:*** I find the correlations in eFigure 1 more meaningful to the estimates in Figure 3.

***Response:*** We agree with this point and thank the reviewer for pointing this out. We have now replaced Fig 3 in the main body with S1 Fig.

***Comment 8:*** The estimates presented in eTable 1 are not how estimates are usually presented. For most readers, much more interpretable estimates would be changes in test scores in metric or standard deviation units for a one-standard deviation increase prior test score. This was done for GPA in the previous paper.

***Response:*** We have decided to remove S1 Table and S1 Fig (former Fig 3) since they contain very similar results to the correlations in Fig 3.

***Comment 9:*** There’s no need for confidence intervals in eTable 1. The estimates and two confidence limits are exactly the same. They just clutter the table.

***Response:*** Please see comment #8 above.

***Comment 10:*** The three cited studies (25-27) are not very relevant to the paper. I found the choice of papers a bit odd; all 3 are highly econometric.

***Response:*** We agree with this point. We have removed the three cited studies (25-27) and replaced them with the following studies:
- Boman B. The influence of SES, cognitive, and non-cognitive abilities on grades: cross-sectional and longitudinal evidence from two Swedish cohorts. European Journal of Psychology of Education. 2023;38(2):587-603. doi: 10.1007/s10212-022-00626-9.
- Marks GN. Cognitive ability has powerful, widespread and robust effects on social stratification: Evidence from the 1979 and 1997 US National Longitudinal Surveys of Youth. Intelligence. 2022;94:101686.
- Marks GN, O'Connell M. Inadequacies in the SES–Achievement model: Evidence from PISA and other studies. Review of Education. 2021;9(3):e3293.

***Comment 11:*** Relatedly, why is the previous study of Danish National Test Program mentioned in the Discussion? The Introduction should provide a summary of the results and limitations of the previous study. The reference should provide a link to the study https://childresearch.au.dk/fileadmin/childresearch/dokumenter/2018_1_2.pdf.

***Response:*** We thank the reviewer for pointing this out. We have now included a description of the previous study of the Danish National School Test Program in the introduction. We further specify what additional findings are reported in our study, and how these contributes on top of the previous description of the Danish National School Program. We have also provided a link to the study in the reference.

***Comment 12:*** The paper would be more interesting if university entrance or tertiary course status were included in the measure of educational attainment, not just completing high school.

***Response:*** We agree with the reviewer that this would be an interesting measure to include. However due to the recency of the Danish National School Test Program (2010-2019), this study was only able to track educational attainment within five years after the children graduated from lower secondary school. We have discussed this in the limitations section page 18:

*Furthermore, because of the recent completeness of the school tests (2010-2019), this study was limited by follow up time and was therefore able to only track the highest educational attainment of children within 5 years after graduating from lower secondary school. As additional data becomes available from children who participated in the Danish National School Test Program, future studies can examine associations between test performance and later socioeconomic characteristics such as educational attainment and income status.*

Reviewer #3

***Comment 1:*** I think it would be appropriate to mention in the introduction that the results presented in this manuscript resemble those presented by Beuchert and Nandrup (reference 16). (Please note, I think there is a typo in ref. 16 (“Rochester”).)

***Response:*** We thank the reviewer for bringing up this point, which was also raised by Reviewer #2. We have now included a description of the previous study of the Danish National School Test Program in the introduction, including its limitations and what our study adds.

***Comment 2:*** It may also be appropriate to refer to the Ludeke et al. (2021) study (see reference below).

***Response:*** We thank the reviewer for referring to the study by Ludeke et al. (2021). We have now included this study in the discussion section.

***Comment 3:*** The study uses the so-called percentile scores from the national tests. I think it would be appropriate to use the underlying Rasch test scores instead. Since student skills are approximately normal distributed, the difference in underlying skill between 50th and 51st percentile (i.e. in the middle of the distribution) is smaller than the difference between 1st and 2nd percentile. To ease interpretability, the Rasch test scores may be standardized with mean 0 and standard deviation 1, which is common in much of the literature using the national test scores.

***Response:*** We agree with the reviewer that the underlying skill in the middle of the percentile distribution is smaller than in the ends, which indeed is a weakness of using percentile scores. We use percentile scores because our study is targeted towards health clinicians and researchers in epidemiology. We believe that this audience may find it challenging to relate standardized Rasch test scores to their research and practice and would find percentile-scores more meaningful. We therefore hope that the reviewer will accept our choice to report percentile scores rather than Rasch Scores or standardized Rasch Scores. We added the following in the discussion, page 18:

*Moreover, the difference in underlying skill between the 50th and the 51st percentile (i.e. in the middle of the distribution) is smaller than the difference between the 1st and 2nd percentile which is a limitation of using percentile test scores compared to Rasch Scores.*

***Comment 4:*** I think it would be more accurate to refer to the test in Danish “reading (Danish)” rather than “language (Danish)” since the tests only assess reading and not verbal language skills.

***Response:*** We agree with the reviewer, and we now refer to the test as “reading (Danish)” and corrected this throughout the manuscript, and all figures and tables.

***Comment 5:*** The Danish Test program was introduced by law earlier than 2010 – but in effect from 2010 (see formulation p. 5)

***Response:*** We thank the reviewer for highlighting this. We have now changed the formulation to reflect that the Danish Test program was implemented in 2010. Please see the manuscript, page 5:

*The Danish National School Test Program was implemented in 2010 as a mandatory individual evaluation tool for children attending public schools.*

***Comment 6:*** Page 11 says that test scores were generally higher in the late years, which leads the authors to speculate that this may be due to “teach to the test”. I do not think this is in consistent with results presented in Table 1 which show that test scores increased from 2010 to 2014 (reading) or 2015 (math) and then declined until 2019.

***Response:*** We agree with the reviewer that our discussion on “teach to the test” is not consistent with the results in Table 1. We have now revised the section in the discussion to reflect that test scores were only increasing from 2010 to 2014/2015.

Discussion, page 17:

*Finally, we found that test scores were generally higher in the late years of the test program compared to the earlier years. We speculate that this general increase in test scores may reflect a potential drawback of standardized testing, wherein teachers “teach to the test”, organizing their teaching around the specific test items found in the Danish National School Test Program.[33] However, test scores did not increase monotonically over the years. Test scores only increased from 2010 to 2014 (reading) and from 2010 to 2015 (math) and subsequently declined until 2019 suggesting that “teaching to the test” may not explain the observation.*
