# Supplementary material for: Utility of data from the Danish National School Test Program for health research purposes: Content and associations with sociodemographic factors and higher education
Source: PLoS One. 2024 May 1;19(5):e0302472. doi: 10.1371/journal.pone.0302472 (PMC11062538; doi:10.1371/journal.pone.0302472)
Supplement: Attachment — Submitted filename: Response to Reviewers.docx [file pone.0302472.s006.docx]

Dear Dr. Daniel Ramskov and editorial staff,

Thank you for sending us the comments and feedback from the editors and reviewers. We thank everyone for taking the time to read our revised manuscript and providing additional valuable input. We appreciate that the peer-reviewers found the changes appropriate. Please find below a detailed point-by-point response to all comments (the editors' and reviewers' comments in black, our replies in blue). The page numbers mentioned are based on the clean version of our manuscript.

# Journal Requirements

***Journal Requirement #1:*** Please review your reference list to ensure that it is complete and correct. If you have cited papers that have been retracted, please include the rationale for doing so in the manuscript text, or remove these references and replace them with relevant current references. Any changes to the reference list should be mentioned in the rebuttal letter that accompanies your revised manuscript. If you need to cite a retracted article, indicate the article’s retracted status in the References list and also include a citation and full reference for the retraction notice.

***Response:*** We have reviewed the reference list and ensured that it is complete and correct.

Reviewer #3

***Comment 1:*** The abstract says that 77.8% of eligible children complete one or more tests. I don’t think it is correct to say that children in independent schools and special needs schools are eligible for the test. I suggest that it says 77.8% of the full population, or – even better – how state both how many of the eligible children (in public schools) complete, and how many of the population of all children compete. This will indicate to researchers, that the test program is of very high quality in terms of response rate (but not necessarily generalizable to children in independent schools). It would also help to highlight one of the contributions of this study relative to Beuchert & Nandrup (ref. no. 5), namely the analysis of non-respondents.

***Response:*** We thank the reviewer for bringing up this point. We agree that this is an important distinction. We have now modified the abstract and results section accordingly.

Please see abstract, page 3:

Among the full Danish population of 1,137,290 children (51.3% male), 960,450 (84.5%) children attended public schools. There were 885,360 children who completed one or more tests in reading or mathematics (test participation was 77.8% for the entire population, and 92.1% for children in public schools).

Results, page 9:

Furthermore, in the subset of children born between 1997 to 2009 who attended a public school (n = 743,010), the proportion with missing tests was even lower (n = 9,380; 1.3%).

***Comment 2:*** The same comments is relevant for the last paragraph in the introduction (p. 4)

***Response:*** We have now added this to the introduction as suggested, page 4:

Our study provides novel, descriptive evidence of the association between demographic and socioeconomic characteristics and performance in the national tests from 2010 to 2019 as well as with later educational attainment, and insights into the participation and non-participation patterns.

***Comment 3:*** First line on p. 8 says that participation is voluntary for independent and special needs schools. I may be wrong, but I do not think independent schools are allowed to use the tests at all. Please double check.

***Response:*** Our understanding is that children in independent and special needs schools cannot participate in the mandatory national tests, but they are allowed to participate in the voluntary national tests (which are conducted at other periods of the year compared to the mandatory tests).

We have now modified the methods, page 8:

Children attending independent and special needs schools may participate in voluntary tests, but this study did not include voluntary school tests.

***Comment 4:*** The first paragraph in the “Population” section (p. 8-9) provides the number of non-completion (22.2%) and the reasons. However it fails to mention the 0.5% with missing information in Danish registers. It is clear from Fig. 1, but confused when reading the text.

***Response:*** We thank the reviewer for pointing this out, and we have now revised this paragraph accordingly.

Please see results, page 8-9:

Among 1,137,290 children (51.3% male) born between 1994 and 2010 who lived in Denmark at six years of age, 885,360 (77.8%) completed one or more tests in reading (Danish) or mathematics from the Danish National School Test Program. A total of 251,930 (22.2%) children did not complete any tests, and in 246,360 (21.7%) children we were able to obtain information from the Danish Education Register regarding their school attendance, and in 5,570 (0.5%) we were not. Likely reasons for children not participating in the tests were because they attended an independent school (n = 152,170; 13.4%), for unknown reasons (i.e., attending public school but were missing a result) (n = 75,090; 6.6%), or attended a special needs school (n = 19,110; 1.7%) (Fig 1).

***Comment 5:*** The discussion mentions the “late for age” result. I agree that these children may have learning difficulties. However, there is also substantial amount of research showing that school starting age itself has a causal effect on learning (see e.g. various papers by Hans Henrik Sievertsen, Helena Skyt Nielsen and Rasmus Landersø.)

***Response:*** Thank you. We have now included this in the discussion, and added the references [33, 34, 35].

We have modified the following in the discussion, page 17:

Children who participated in a test “late for age” had an 18-point lower test score compared to children who participated at expected age. Some evidence indicates that there is a direct effect of school starting age on learning outcomes [33, 34, 35], but this finding may also suggest that “late for age” children may be more likely to have pre-existing learning difficulties, which delays school entry or result in children repeating grades.

***Comment 6:*** I think the authors should delete that last paragraph on p. 17, which speculate about teaching to the test for the following reasons: 1) it is only speculative, there is no data to support it. Many other explanations could be relevant, e.g. that teachers or parents have become better at learning children to read 2) as mentioned in my first review, the results do not increase uniformly, which speaks against the teach to test-explanation (which the manuscript now acknowledges, but this makes it unclear why to mention the explanation in the first place) 3) the last sentence suggesting that studies should adjust for calendar-year does not make sense in relation to the rest of the study because it indicates that the test scores are not valid measures of reading skills. If students improved their reading skills (and not just became better at taking the test), adjusting for calendar-year would be a mistake.

***Response:*** We agree with the reviewer, and we have therefore decided to delete the paragraph about “teaching to the test” in the discussion.

We have also removed the reference “Popham WJ. Teaching to the Test? Educational leadership. 2001;58(6):16-21”.

***Comment 7:*** The limitations section now mentions the difference between the Rasch score and the percentile score (p. 18). I suggest that the manuscript presents an explanation for why the study chose to use the percentile score. The authors response to my comment about this, could be a fine explanation to include in the manuscript.

***Response:*** As suggested, we added the following in the discussion, page 18:

Moreover, the difference in underlying skill between the 50th and the 51st percentile (i.e. in the middle of the distribution) is smaller than the difference between the 1st and 2nd percentile which is a limitation of using percentile test scores compared to Rasch test scores. However, we use percentile scores because our study is targeted towards health clinicians and researchers in epidemiology. We believe that this audience may find the percentile scores more intuitive and easier to interpret (e.g. what constitutes a large versus small score difference) than the Rasch scores.
